# Supplementary figures and images for: DNA barcoding for biodiversity assessment: Croatian stoneflies (Insecta: Plecoptera)
Source: PeerJ. 2022 Apr 20;10:e13213. doi: 10.7717/peerj.13213 (PMC9034701; doi:10.7717/peerj.13213)

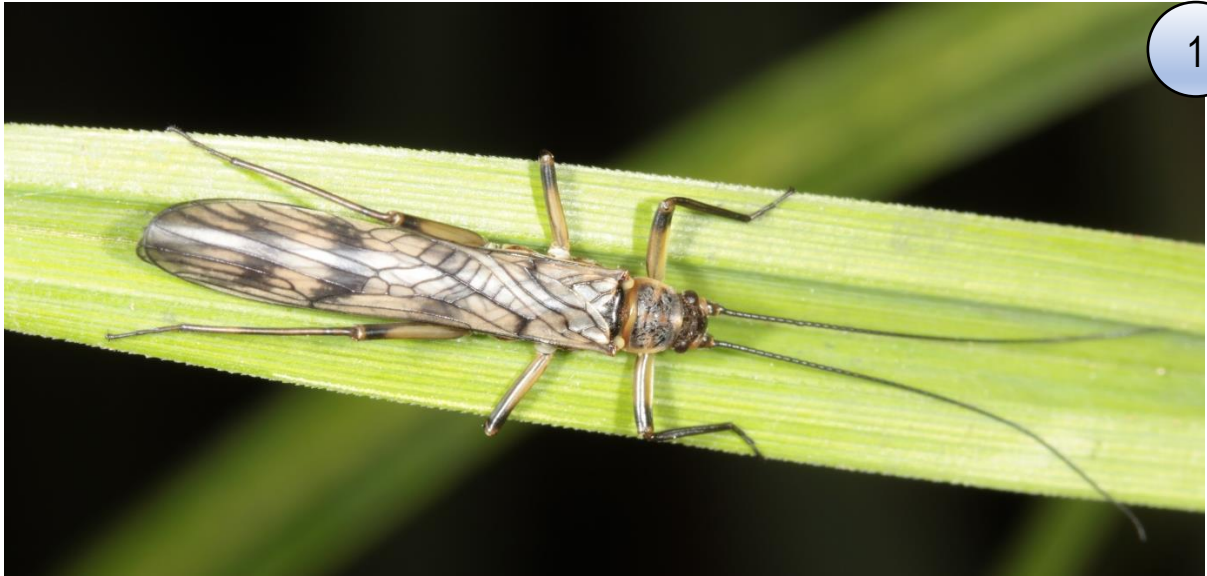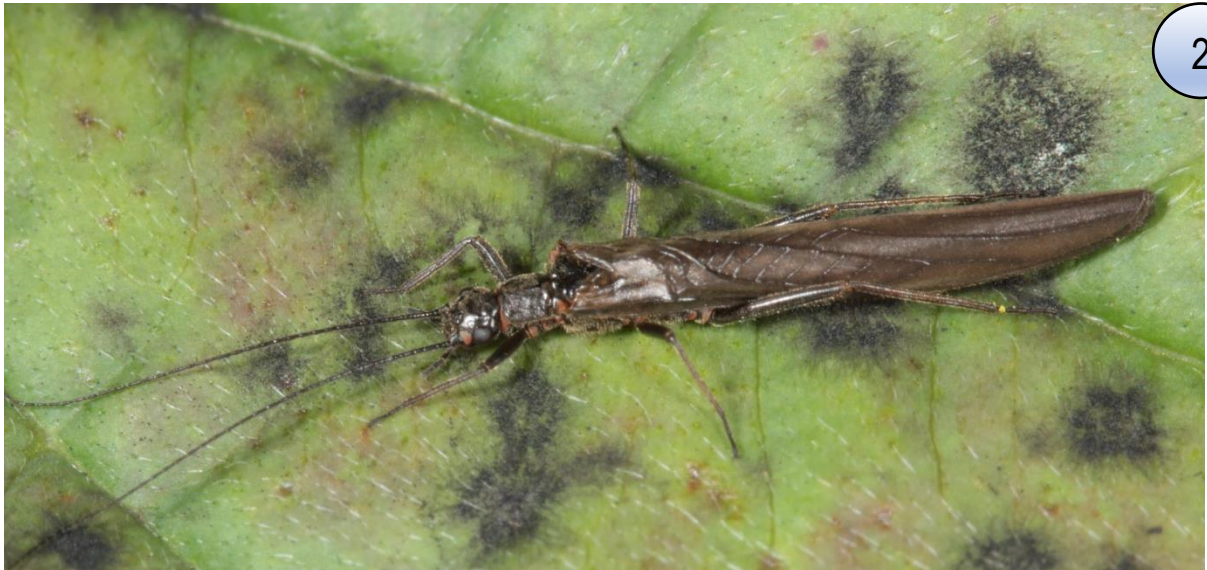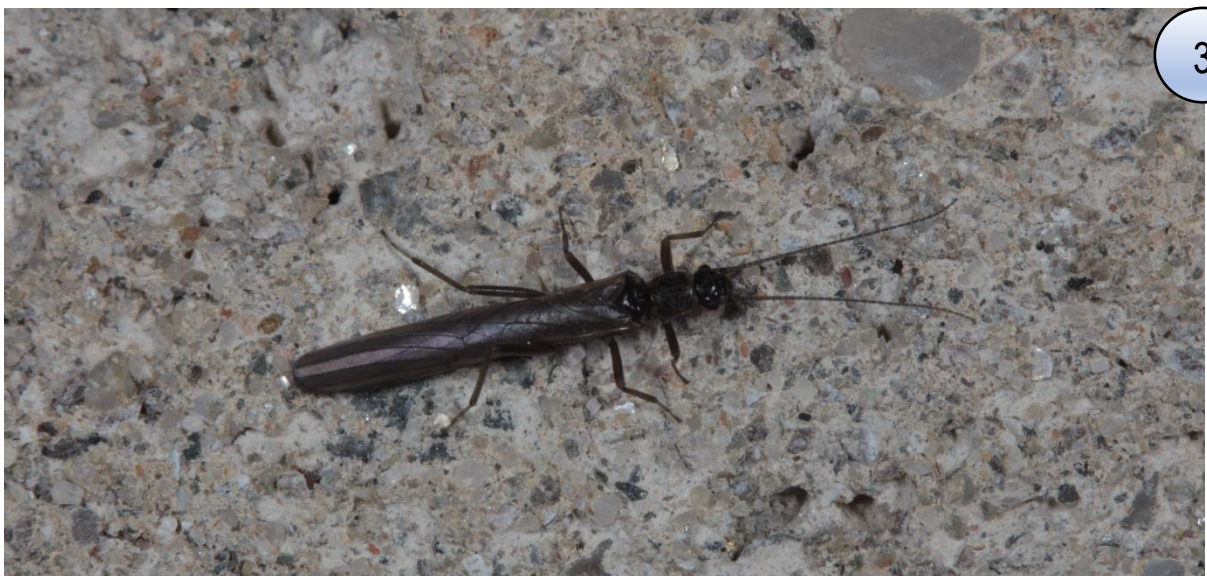

4

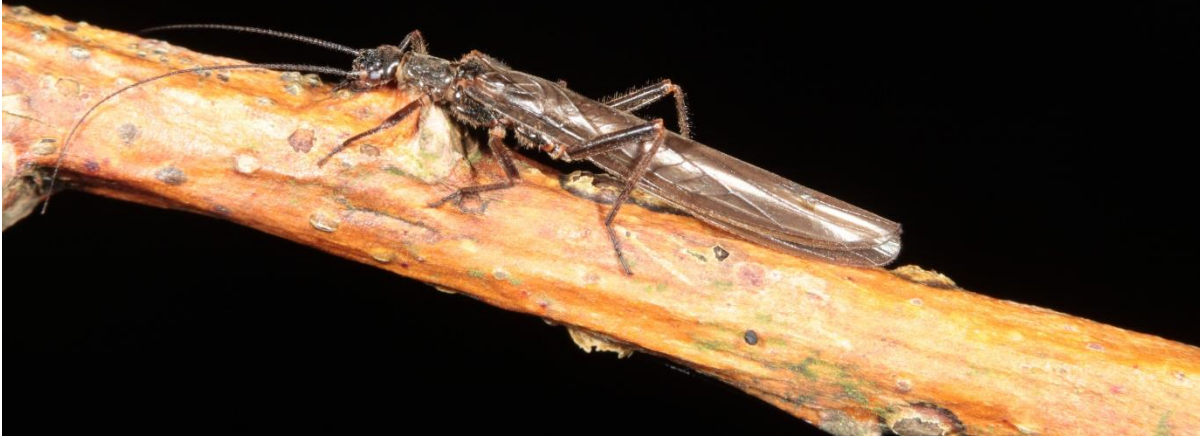

5

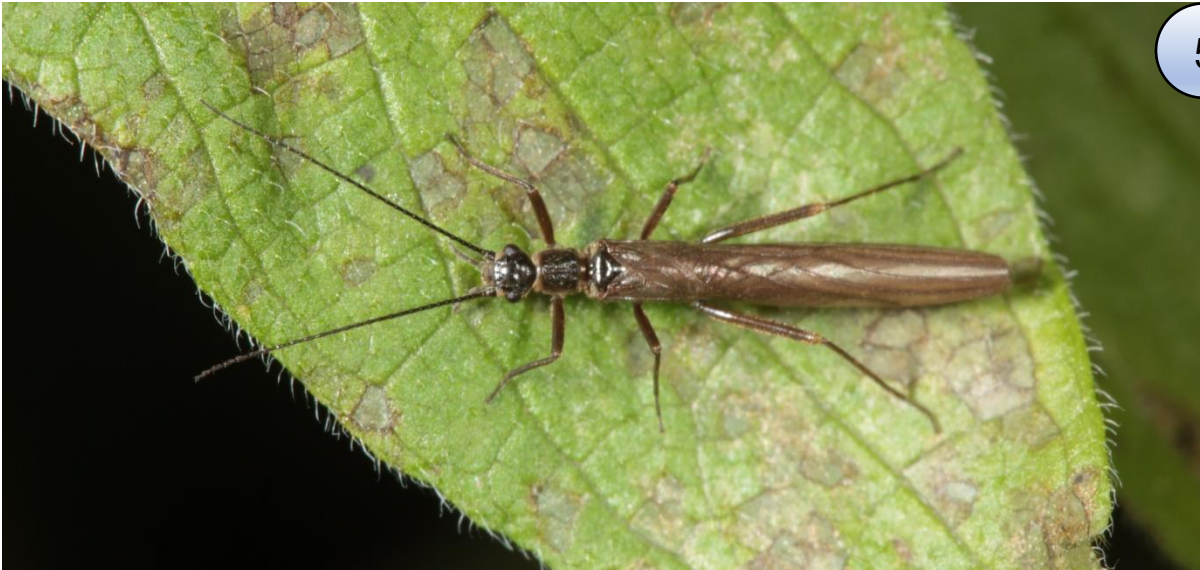

6

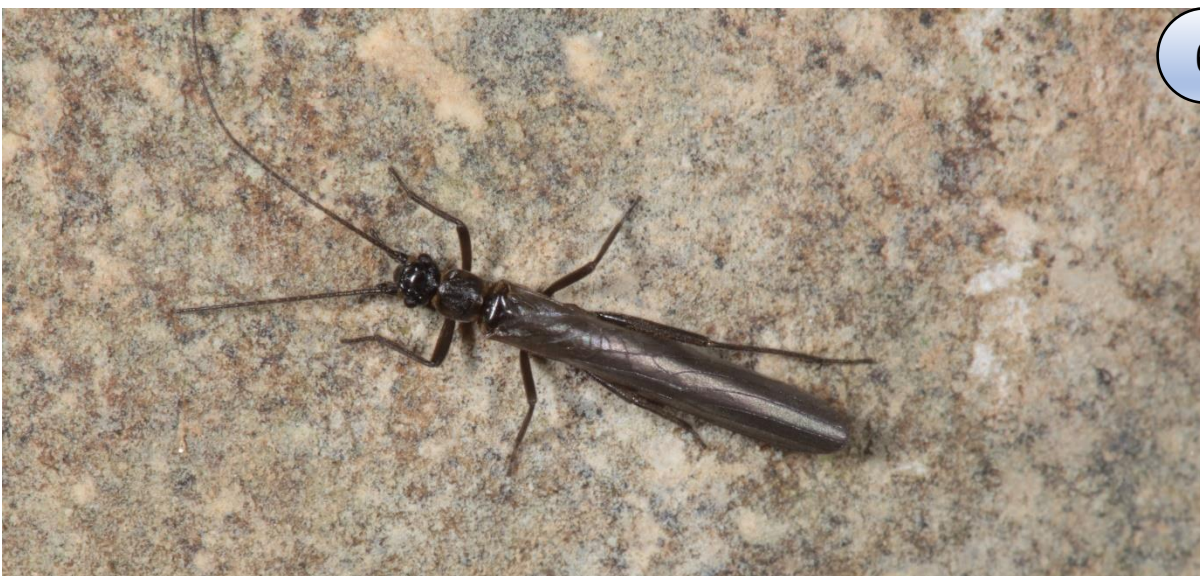

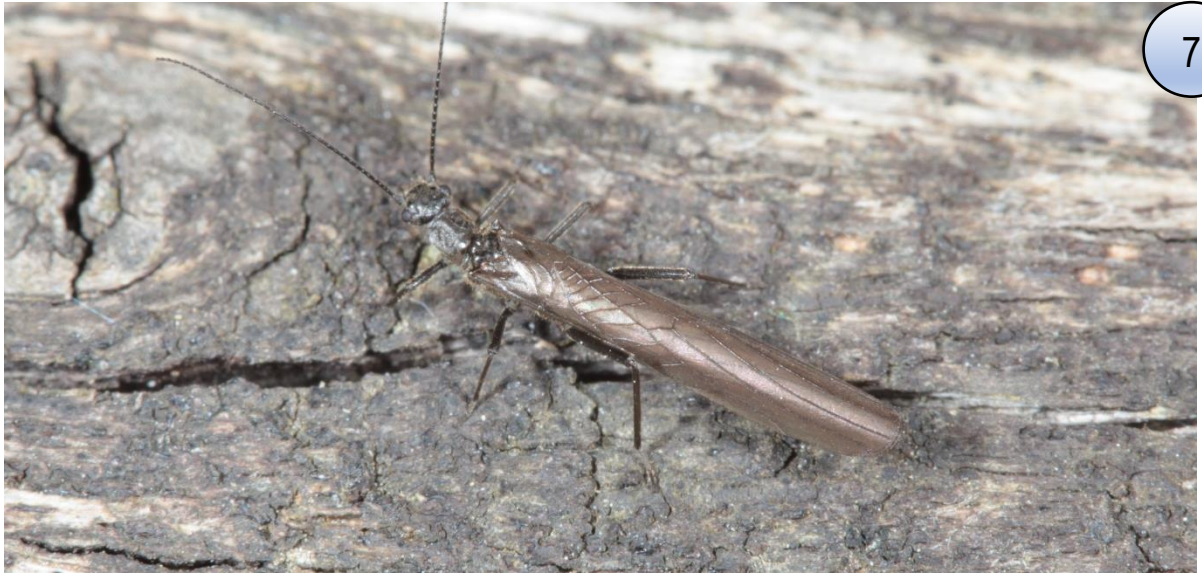

7

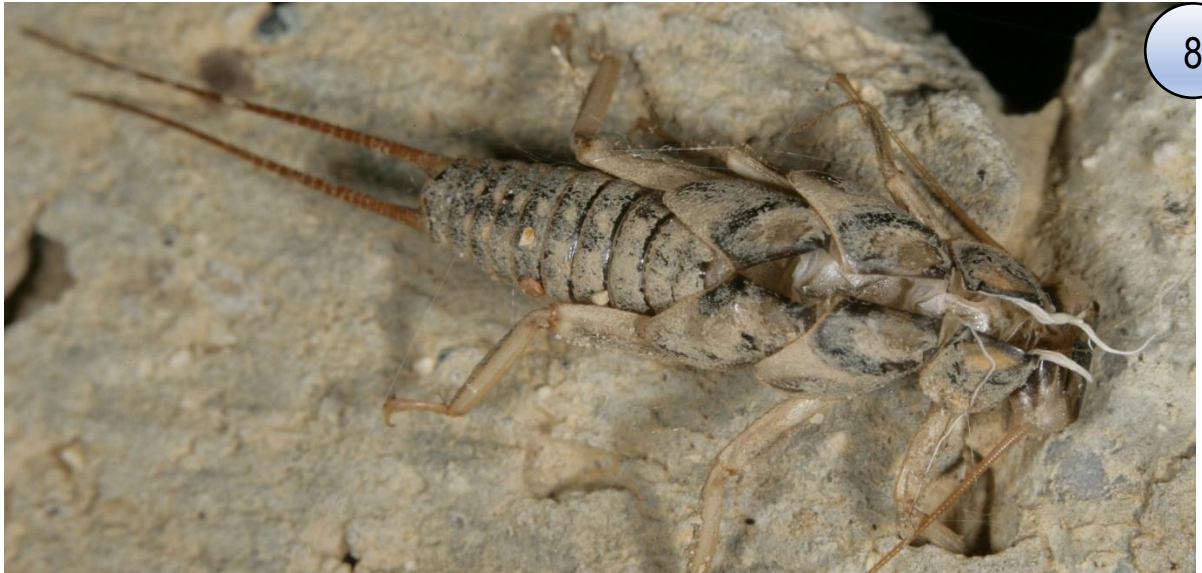

8

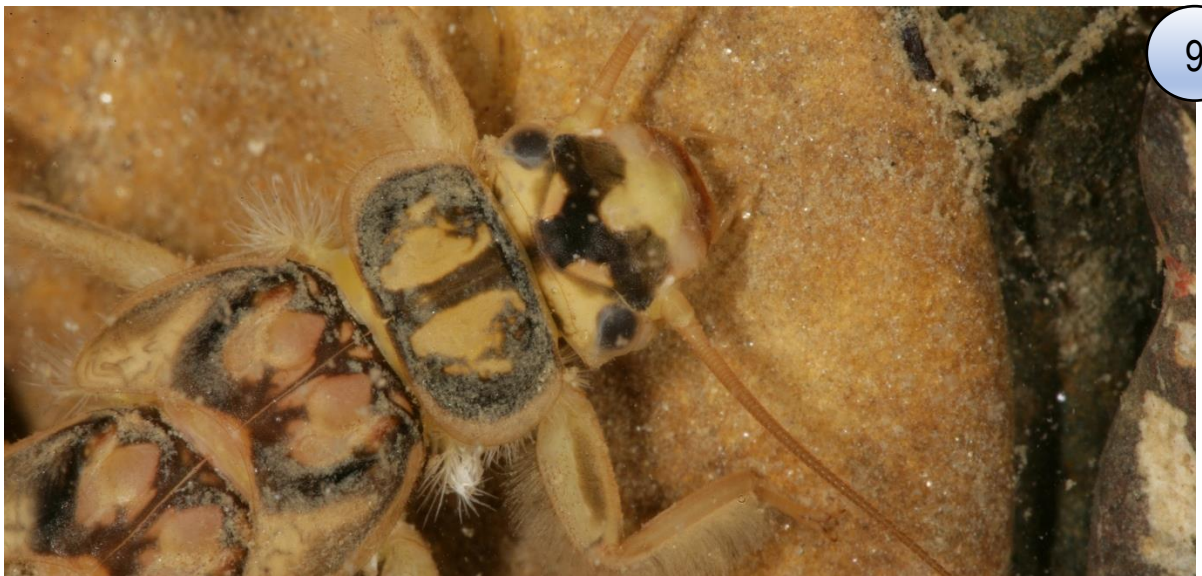

9

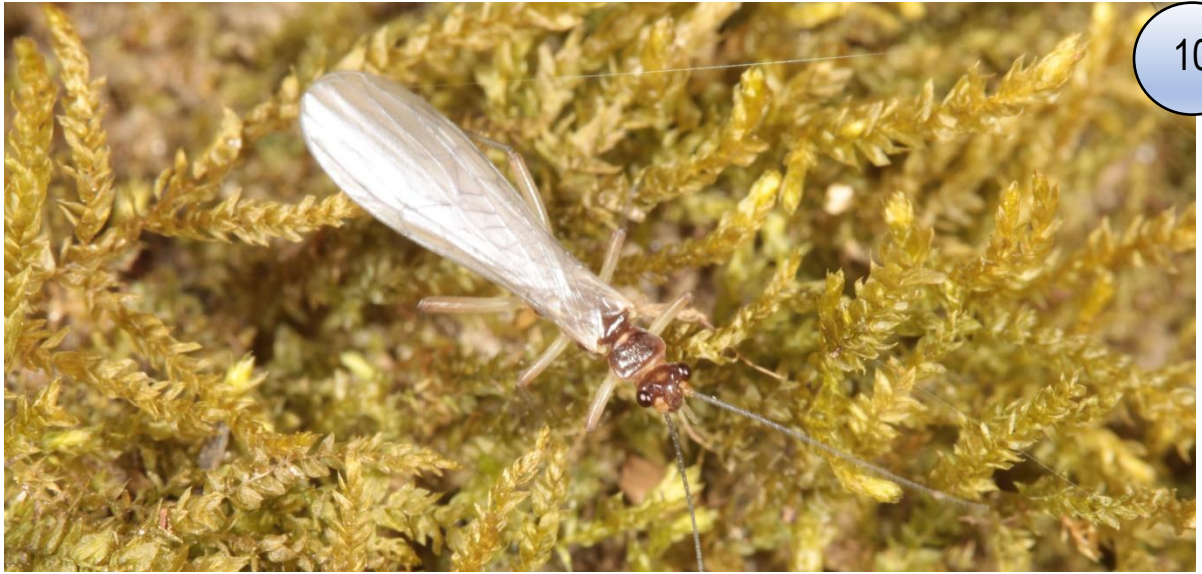

10

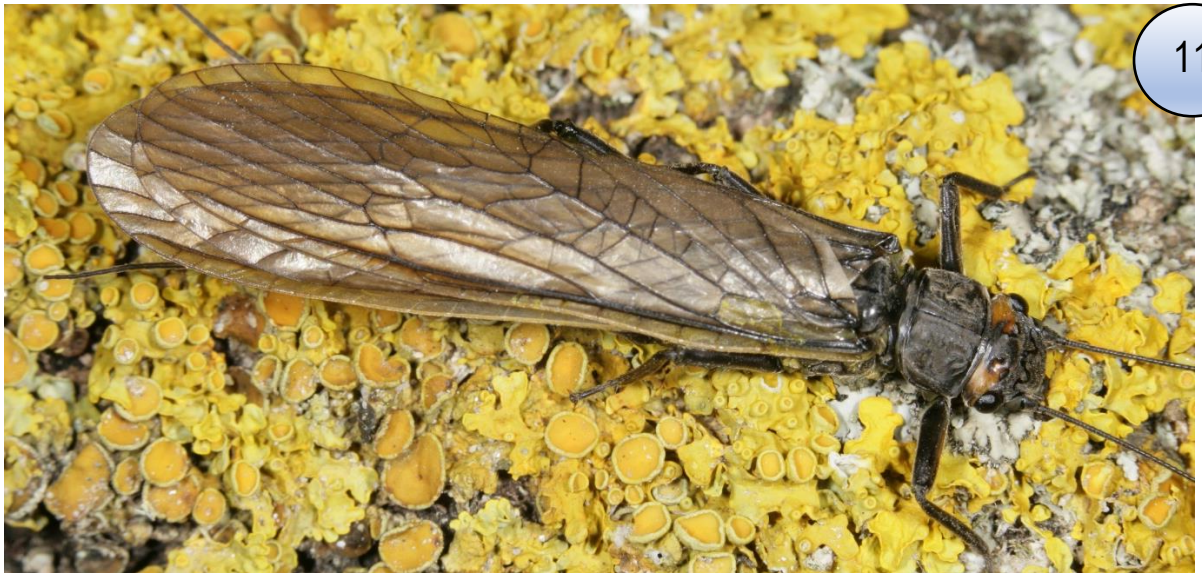

11

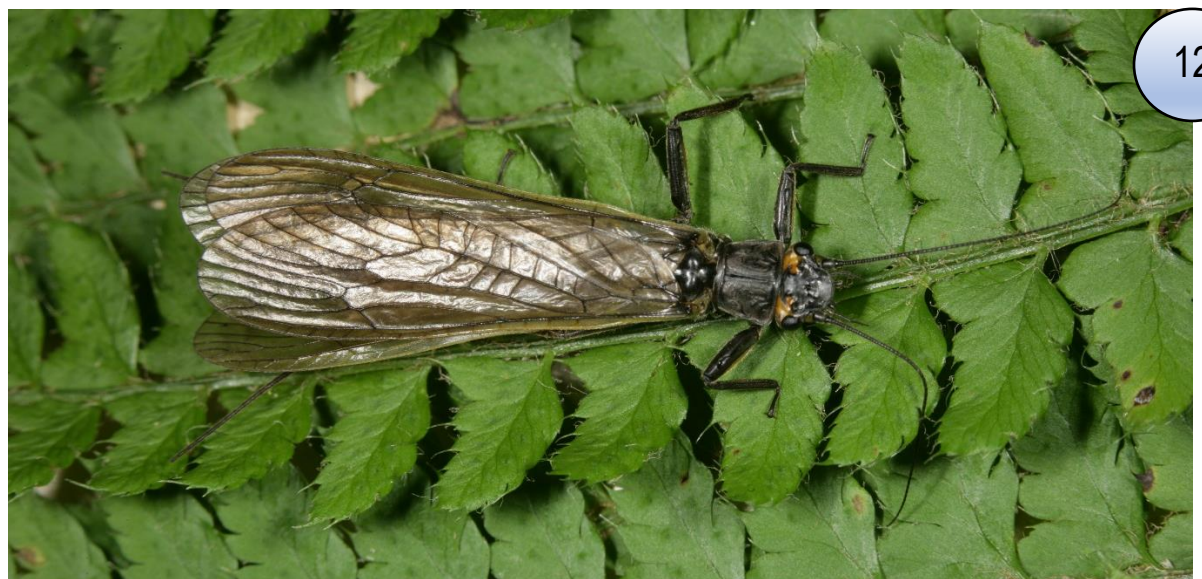

12

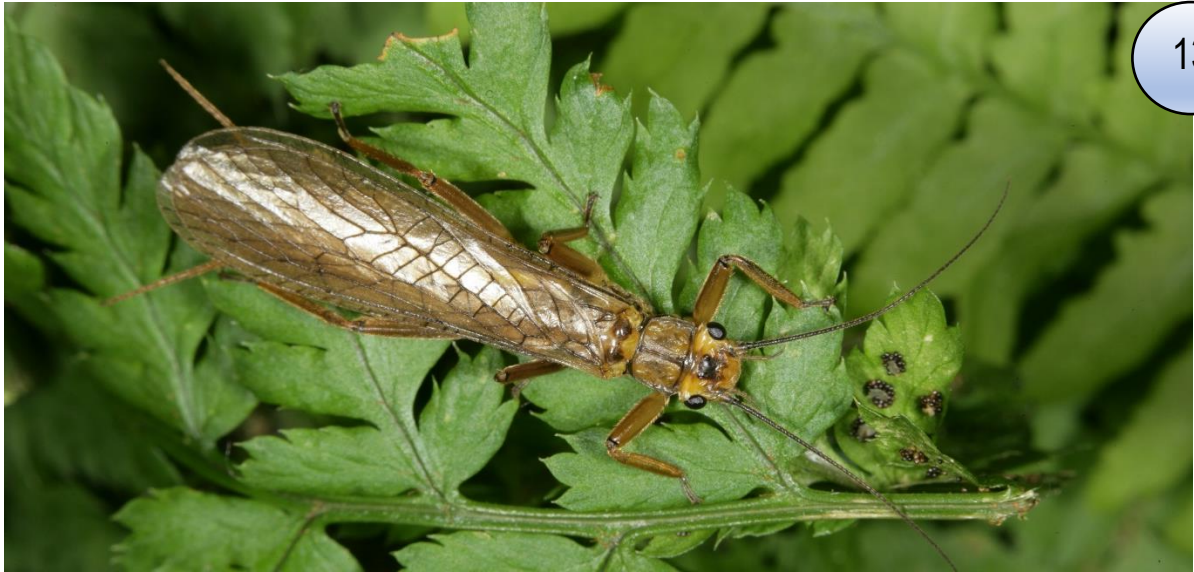

13

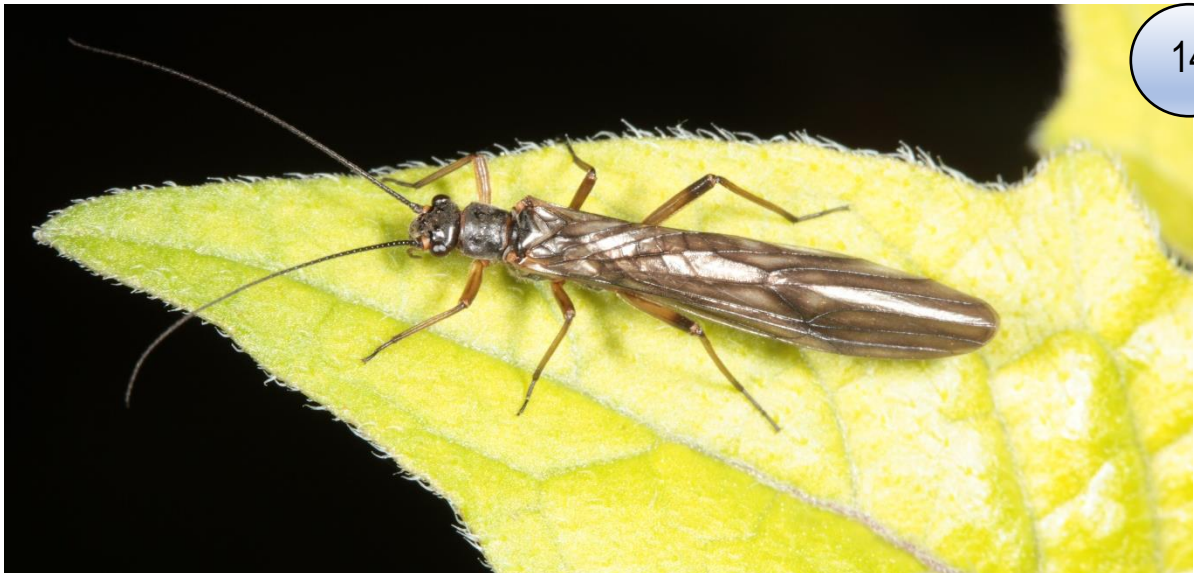

14

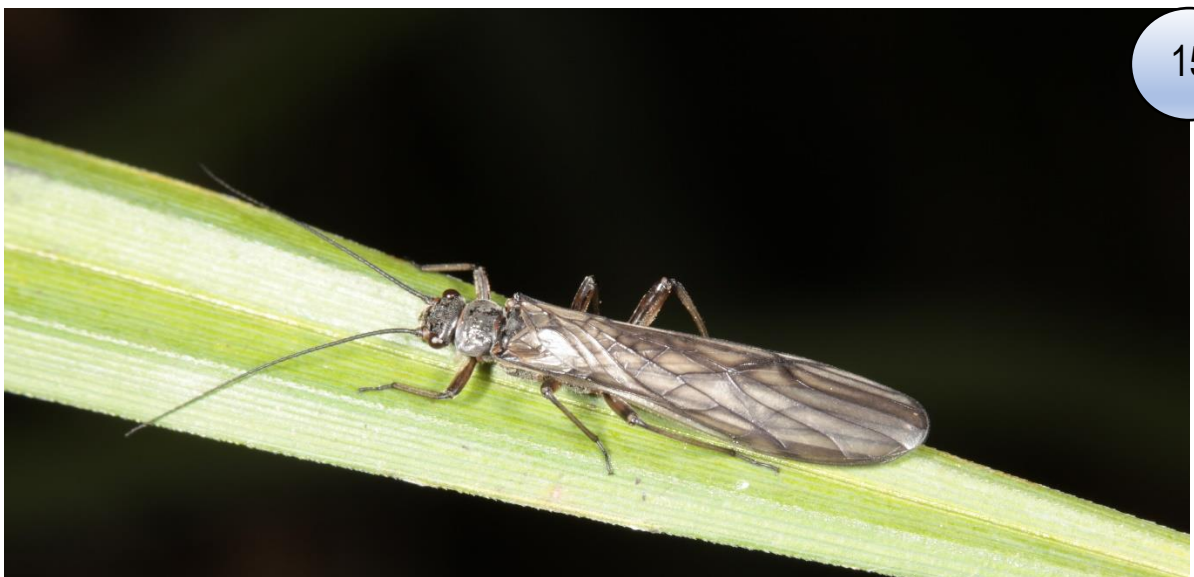

15

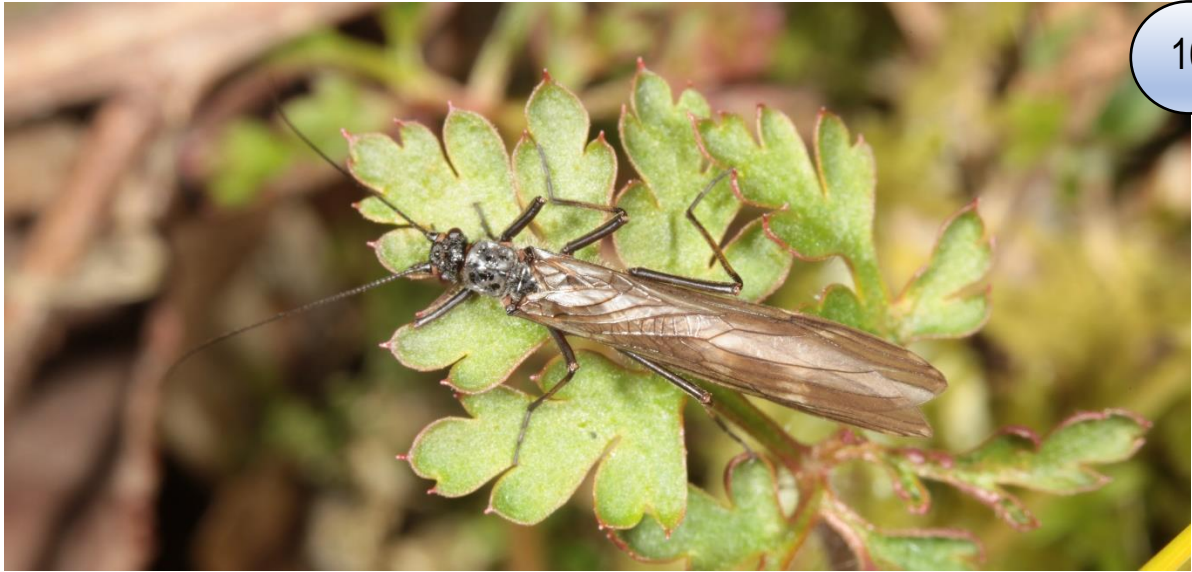

16

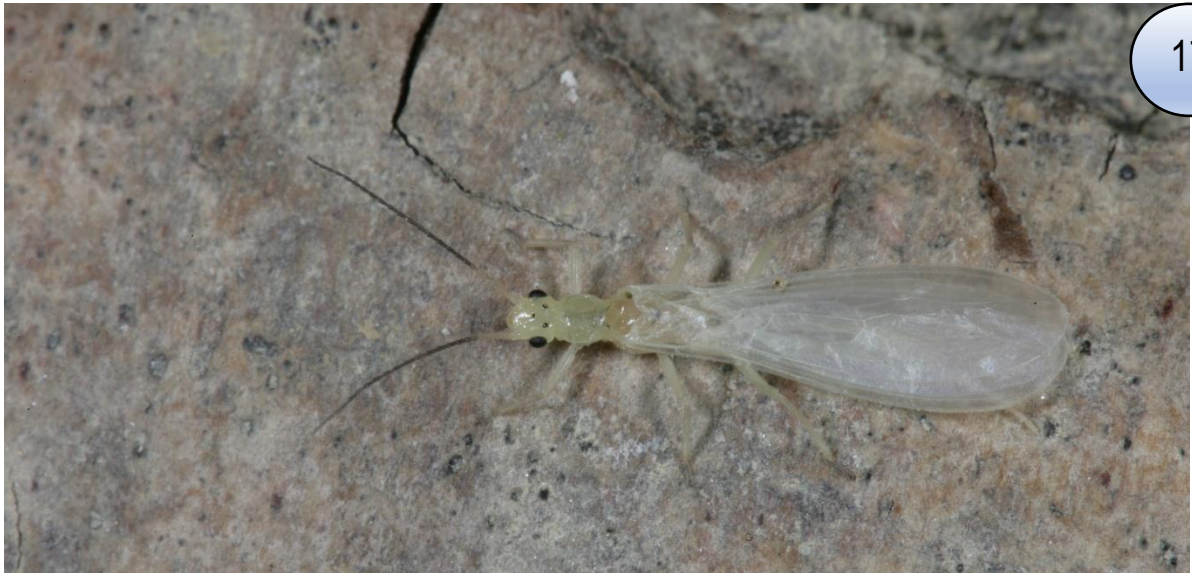

17

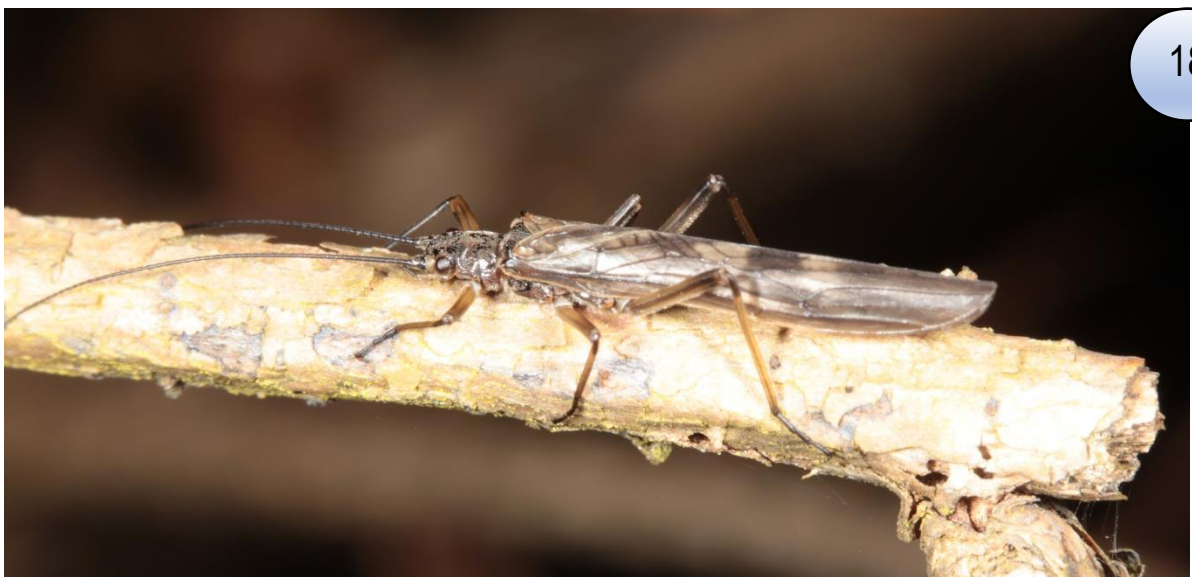

18

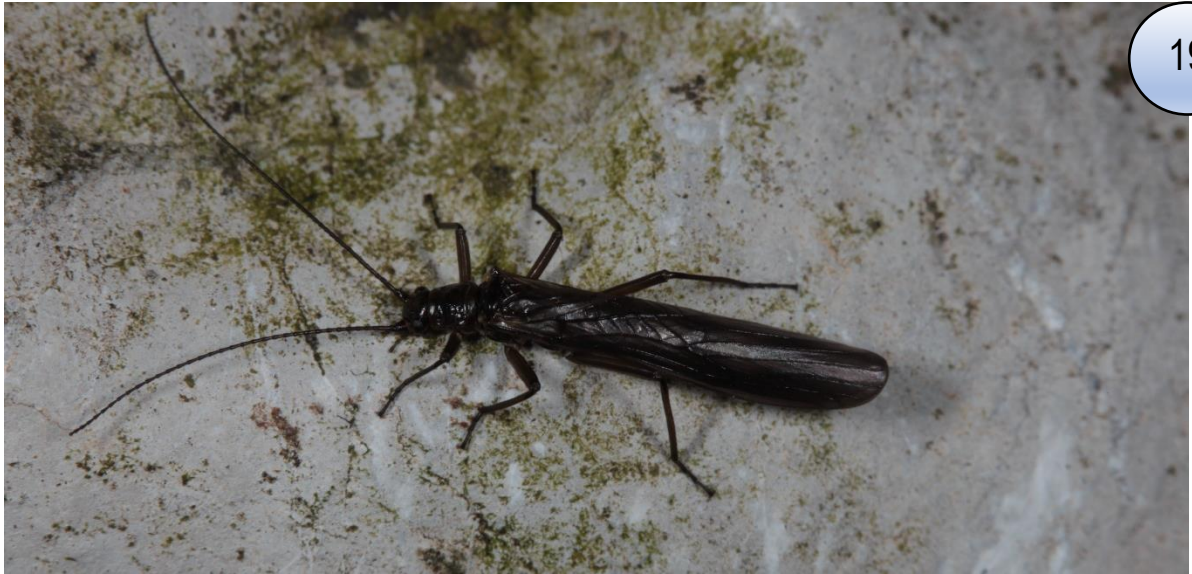

19

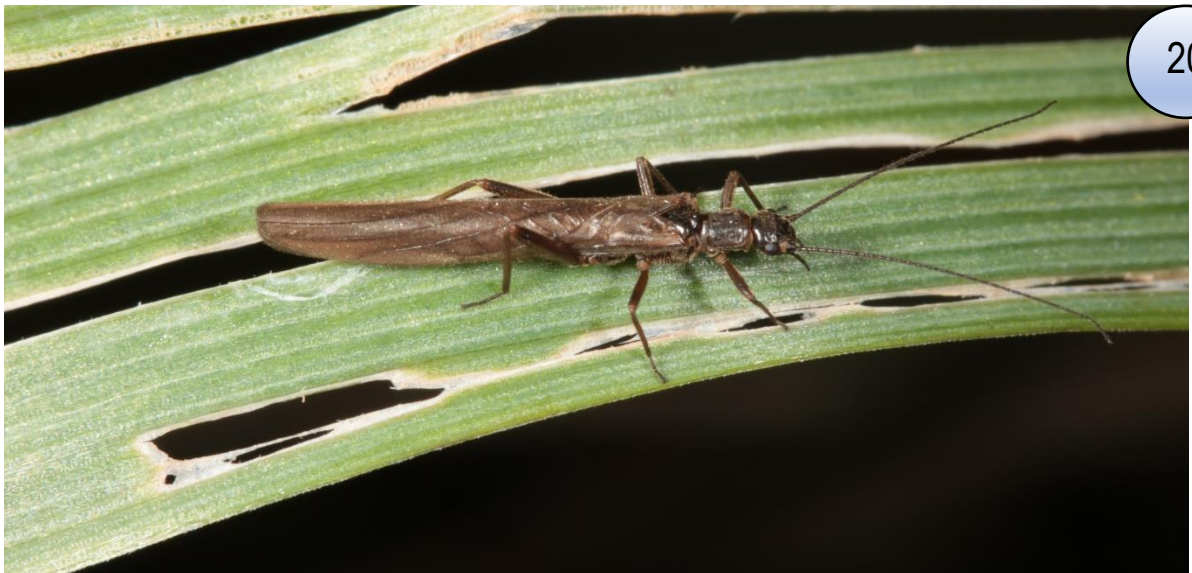

20

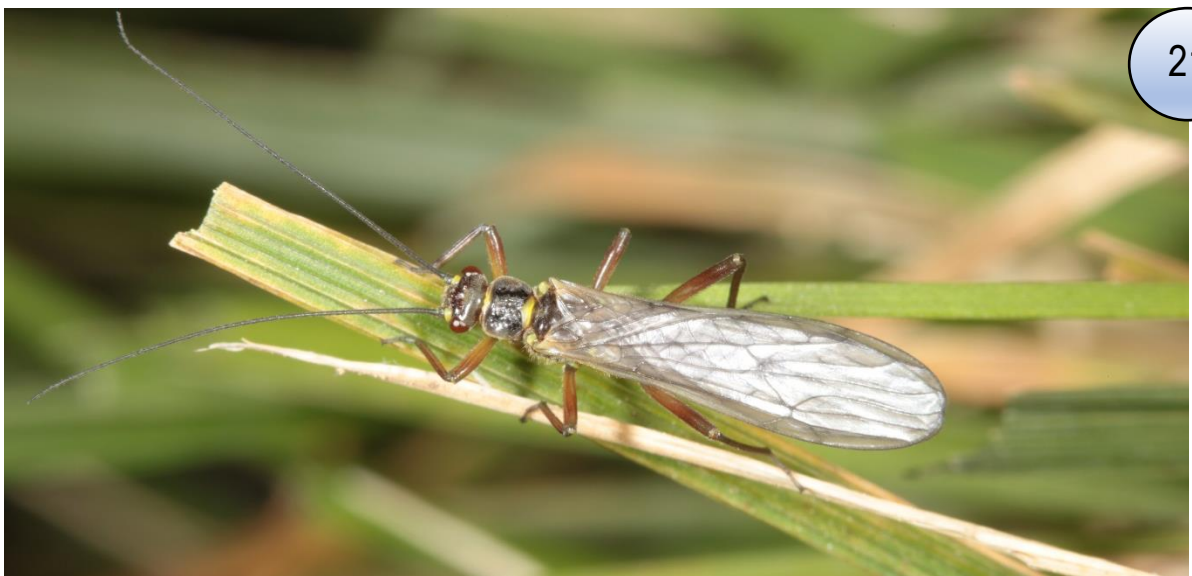

21

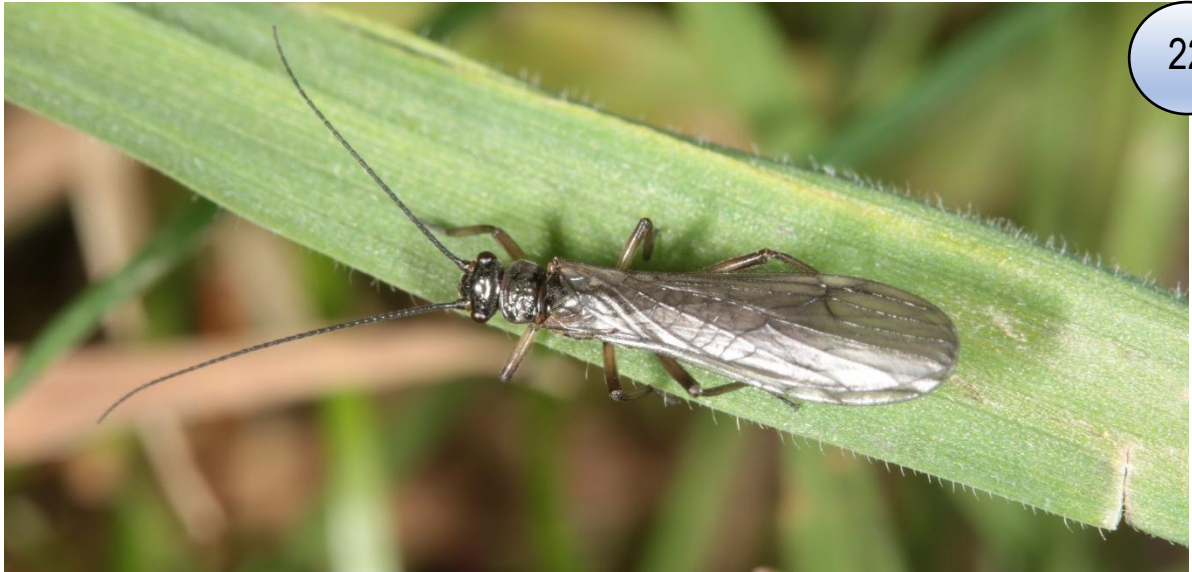

22

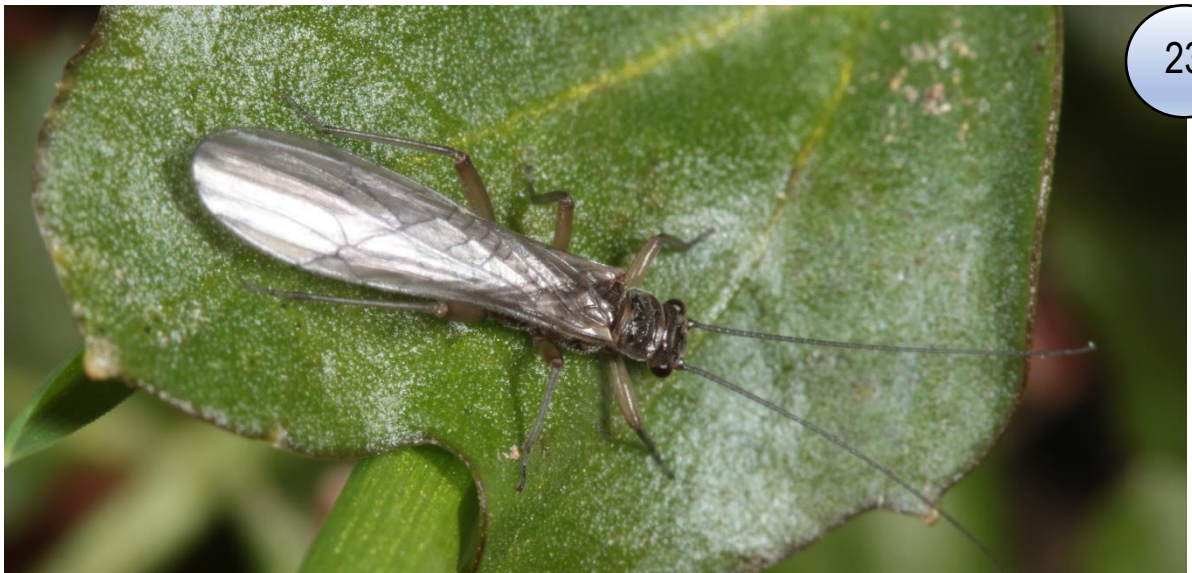

23

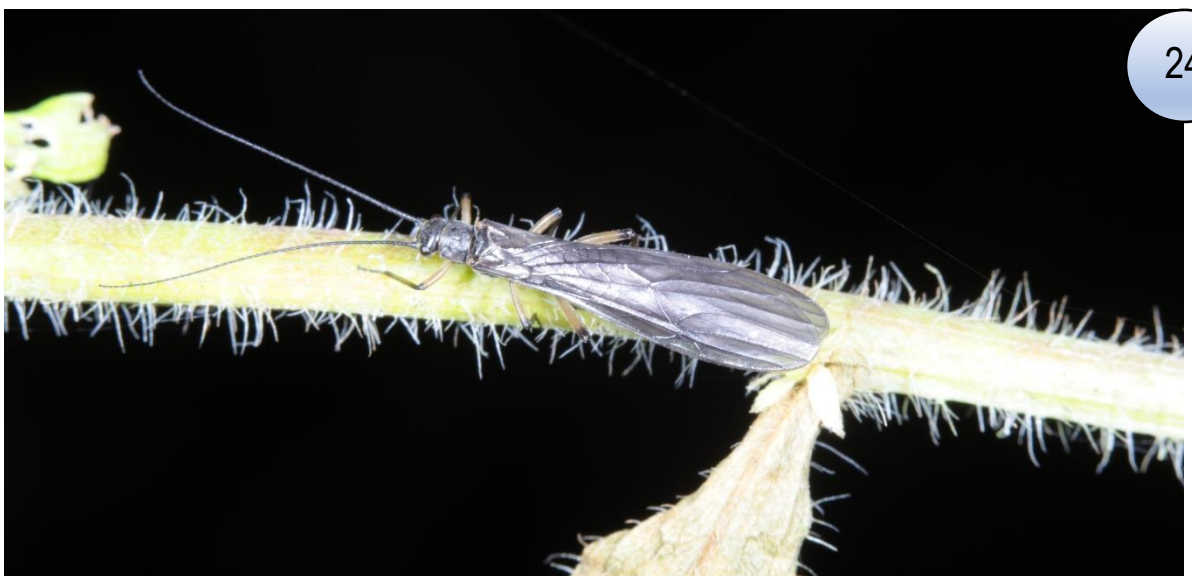

24

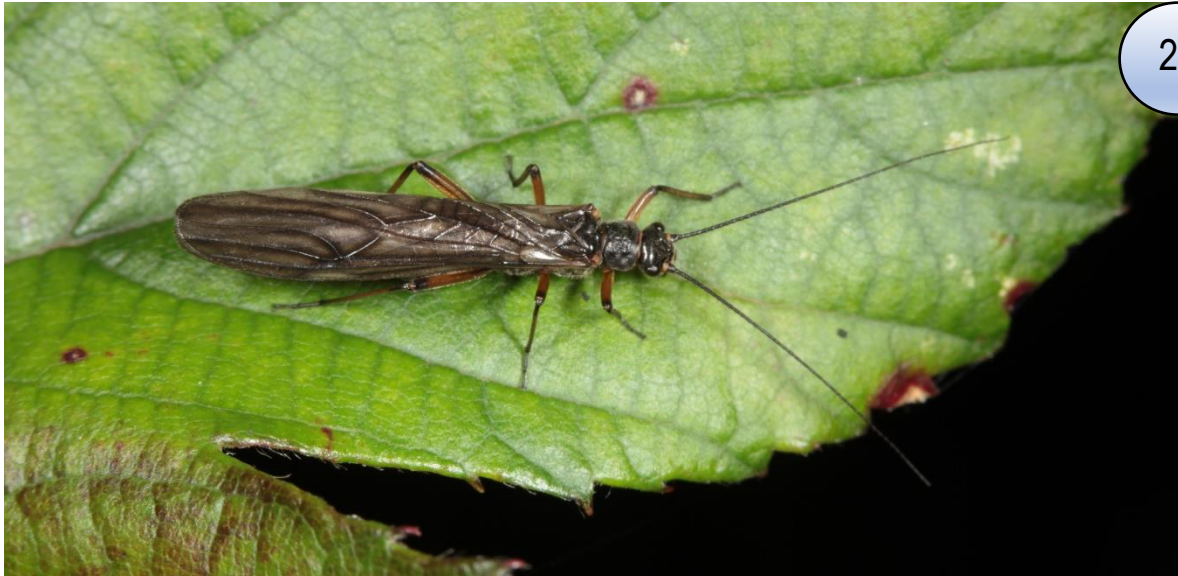

25

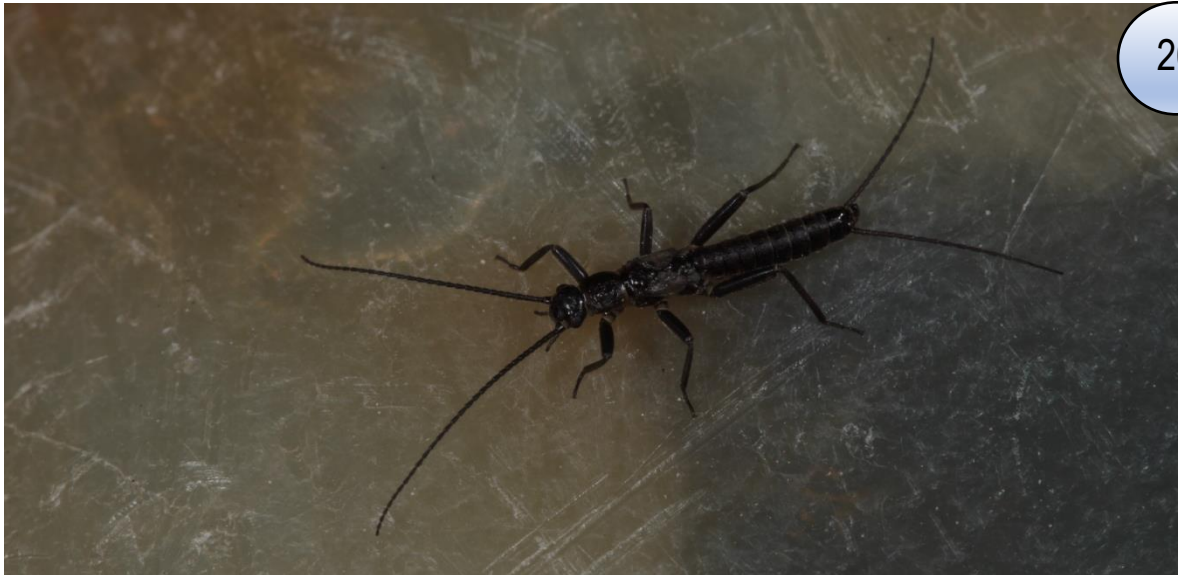

26

Supplement: Supplemental Information 1 — 1. Brachyptera seticornis; 2. Leuctra fusca; 3. Leuctra inermis; 4. Leuctra major; 5. Leuctra mortoni; 6. Leuctra nigra; 7. Leuctra prima; 8. Marthamea vitripennis; 9. Marthamea vitripennis; 10. Nemoura marginata; 11. Perla burmeisteriana; 12. Perla illiesi; 13. Perla marginata; 14. Protonemura hrabei; 15. Protonemura praecox; 16. Taeniopteryx n.sp. CRO-1; 17. Xanthoperla apicalis; 18. Brachyptera risi; 19. Brachyptera tristis; 20. Leuctra albida; 21. Nemoura avicularis; 22. Nemoura dubitans; 23. Nemoura sciurus; 24. Nemurella picteti; 25. Protonomeura nitida; 26. Zwicknia bifrons. Photographs taken by I. Sivec. [file peerj-10-13213-s001.pdf]

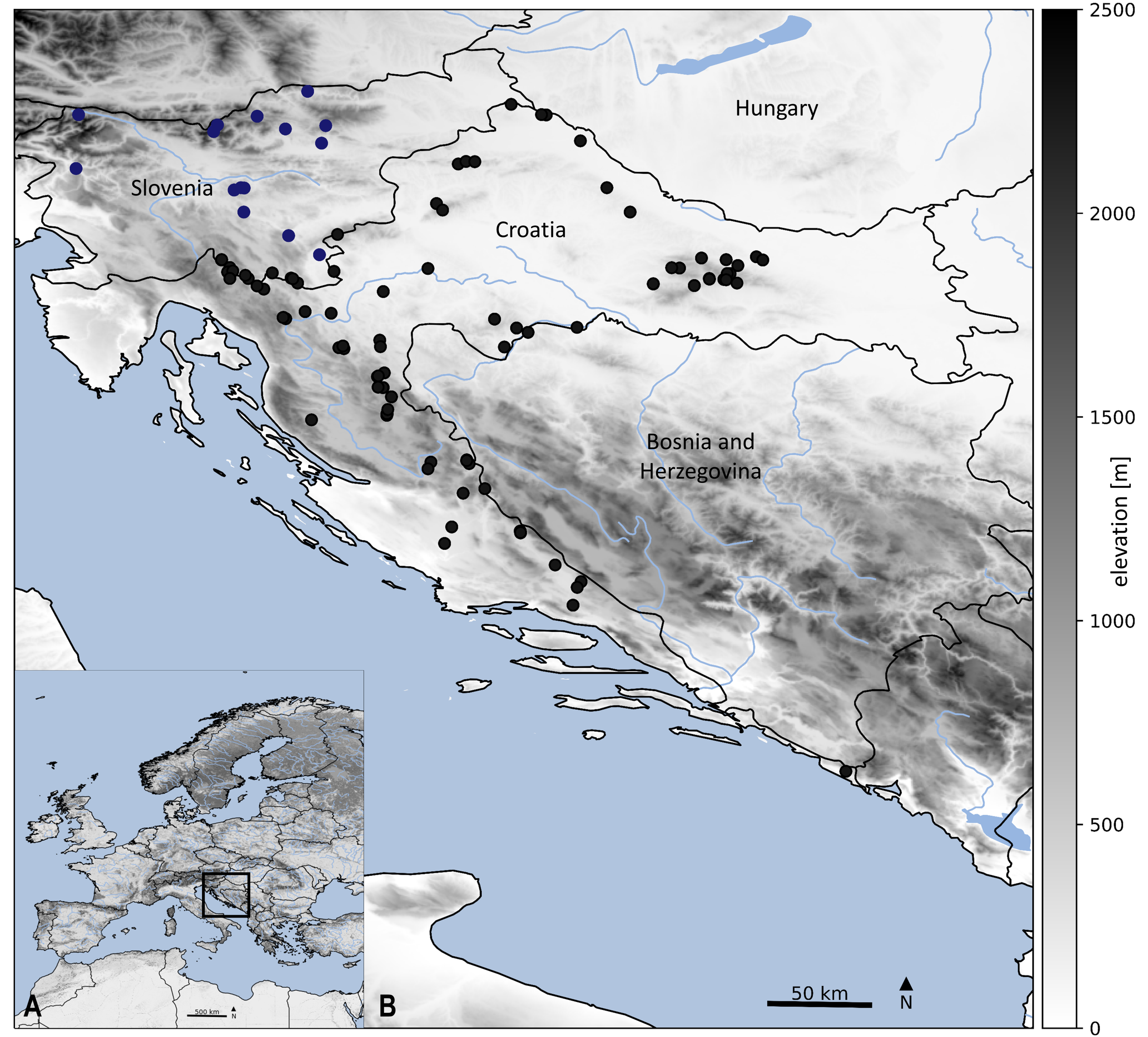

Supplement: Supplemental Information 4 — Details about sampling sites are provided in Table S1. Symbols used on the map: black dots represent localities in Croatia and blue dots additional localities in Slovenia. Main map (B) is an enlarged framed area in the bottom left corner (A). Map is produced with Cartopy package 0.19 in Python with use of elevation data from https://land.copernicus.eu/imagery-in-situ/eu-dem/eu-dem-v1.1. [file peerj-10-13213-s004.png]

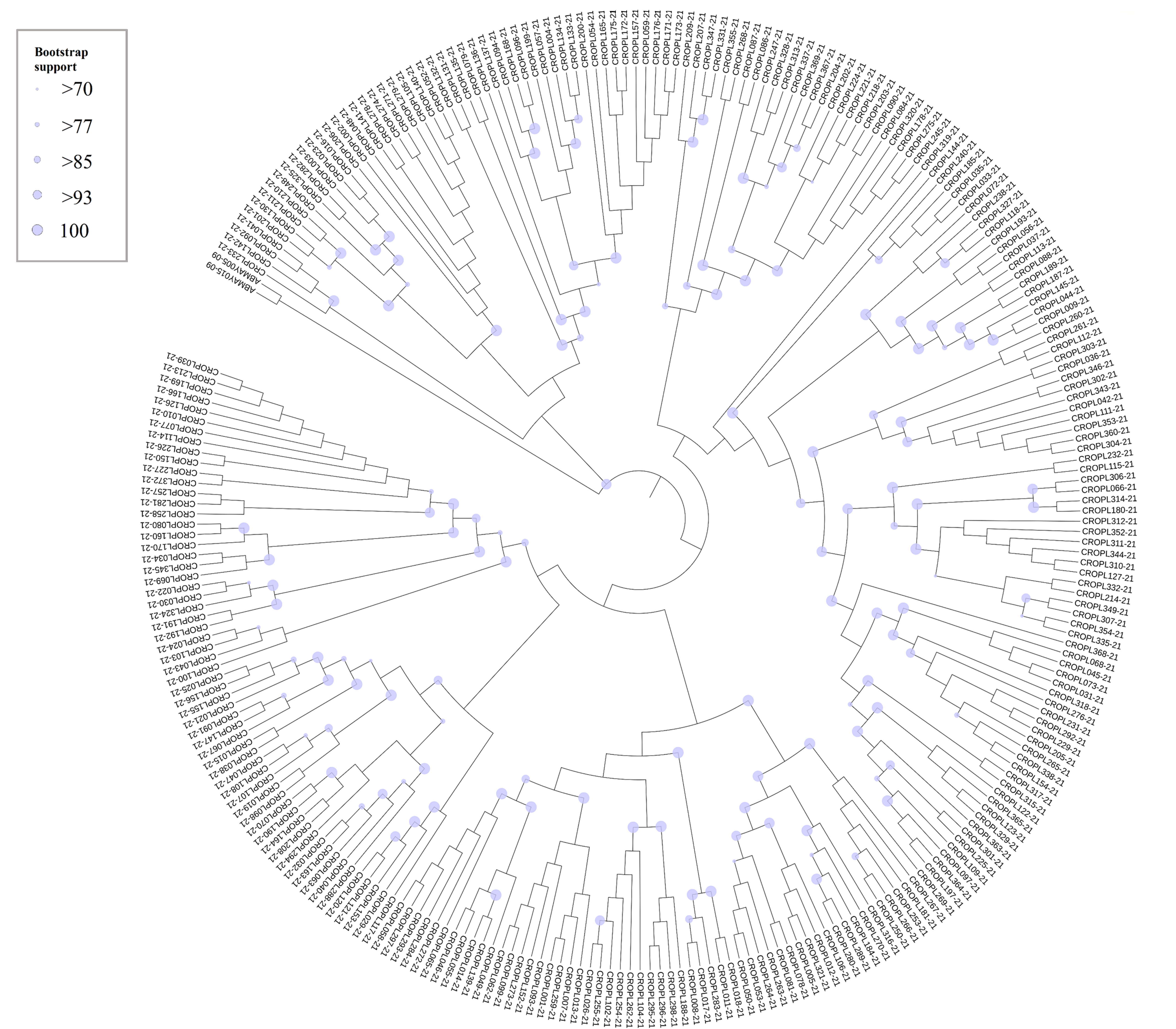

Supplement: Supplemental Information 5 — Size of dots at nodes is proportional to the bootstrap support value. Terminal codes present BOLD IDs, as in Table S1. The tree was annotated in iTOL ver. 5 (Letunic & Bork, 2021) and finished in Adobe Illustrator. [file peerj-10-13213-s005.png]

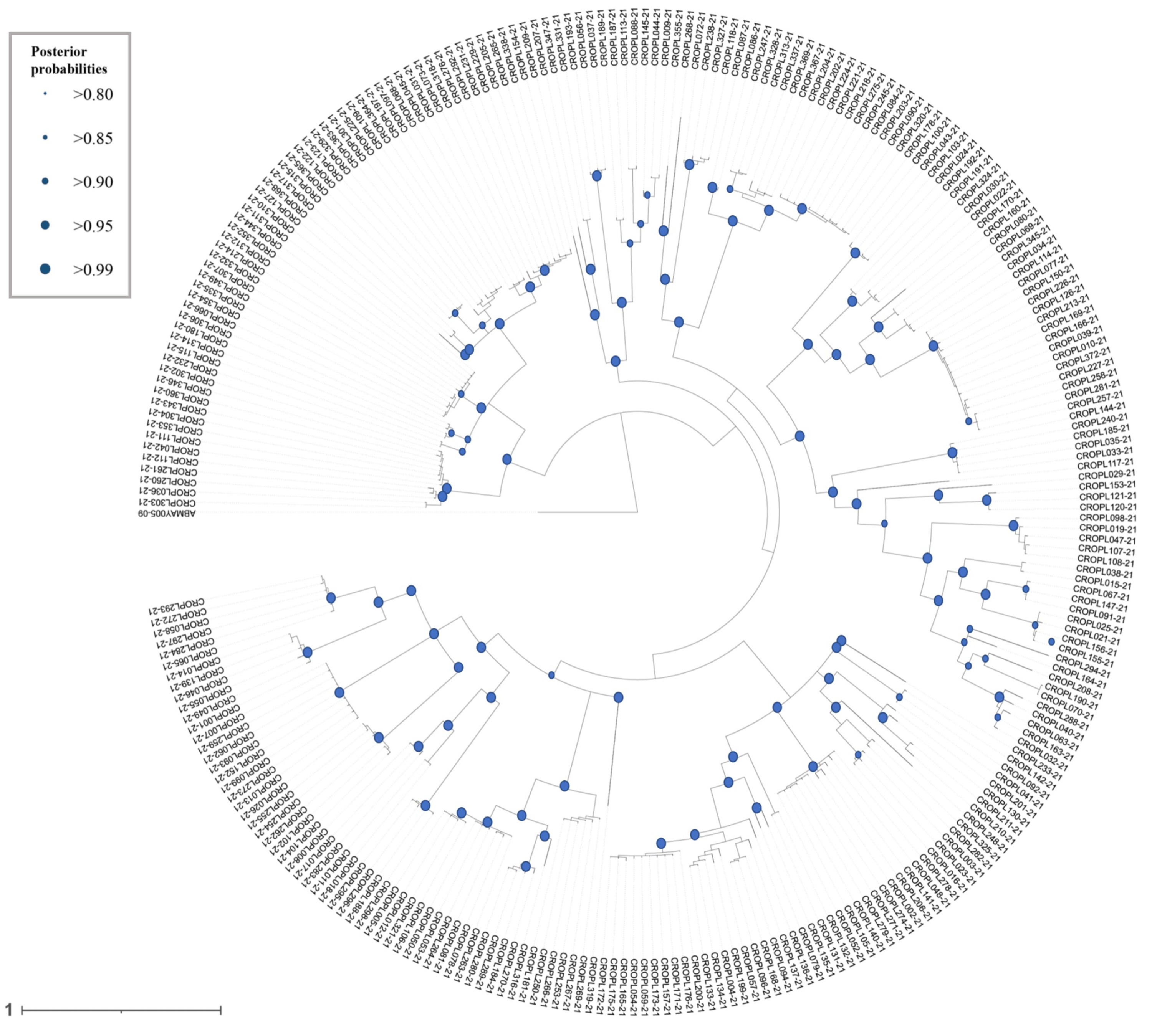

Supplement: Supplemental Information 6 — Size of dots at nodes is proportional to the Bayesian posterior probability (BPP) categories. Terminal codes present BOLD IDs, as in Table S1. The tree was annotated in FigTree ver. 1.4.3 (Rambaut, 2009) and iTOL ver. 5 (Letunic & Bork, 2021) and finished in Adobe Illustrator. [file peerj-10-13213-s006.png]
