# Supplementary material for: DNA barcoding for biodiversity assessment: Croatian stoneflies (Insecta: Plecoptera)
Source: PeerJ. 2022 Apr 20;10:e13213. doi: 10.7717/peerj.13213 (PMC9034701; doi:10.7717/peerj.13213)
Supplement: Supplemental Information 8 [file peerj-10-13213-s008.docx]

| **PCR primers** | (a) LCO-1490/HCO-2198  (b) C_LepFolF/C_LepFolR  (c) MLepF1/LepR1  MLepR1/LepF1 | *Folmer et al., 1994*  *Folmer et al., 1994*  *Hebert et al., 2003a*  *Hebert, Ratnasingham & deWaard, 2003b*  *Hajibabaei et al., 2006* |
| --- | --- | --- |
| **Thermocycling conditions** | (a)  initial denaturation at 95°C for 2 min, followed by 35 cycles of denaturation at 95°C for 30 s, annealing at 50°C for 30 s, extension at 72°C for 1 min, followed by a final extension step at 72°C for 10 min  (b)  initial denaturation step of 95°C for 10 min followed first by 5 cycles of 30 s at 95 °C, 30 s at 45°C and 60 s at 72 °C, then by 35 cycles of 30 s at 95 °C, 30 s at 51°C and 60 s at 72 °C; ended with the final extension step at 72 °C for 7 min  (c)  initial denaturation at 95°C for 2 min, followed by 40 cycles of 40 s at 94 °C, 40 s at 51°C and 30 s at 72 °C; ended with the final extension step at 72 °C for 5 min | |

Folmer O, Black M, Hoeh W, Lutz R, Vrijenhoek R. 1994. DNA primers for amplification of mitochondrial cytochrome c oxidase subunit I from diverse metazoan invertebrates. *Molecular Marine Biology and Biotechnology* **3**:294–299

Hebert PDN, Cywinska A, Ball SL, deWaard JR. 2003a. Biological identifications through DNA barcodes. *Proceedings of the Royal Society of London. Series B: Biological Sciences* **270**(1512):313–321

Hebert PDN, Ratnasingham S, deWaard JR 2003b. Barcoding animal life: cytochrome c oxidase subunit 1 divergences among closely related species. *Proceedings of the Royal Society B: Biological Sciences* **270**:S96–S99

Hajibabaei M, Janzen DH, Burns JM, Hallwachs W, Hebert PDN. 2006. DNA barcodes

distinguish species of tropical Lepidoptera. *Proceedings of the National Academy of Sciences of the USA* **103(4)**:968–971
