# Supplementary material for: DNA barcoding for biodiversity assessment: Croatian stoneflies (Insecta: Plecoptera)
Source: PeerJ. 2022 Apr 20;10:e13213. doi: 10.7717/peerj.13213 (PMC9034701; doi:10.7717/peerj.13213)
Supplement: Supplemental Information 10 [file peerj-10-13213-s010.docx]

* NN – Nearest neighbour

| **Species** | **Mean intra-Sp (%)** | **Max intra-Sp (%)** | **Nearest species** | **NN** | **Distance to NN (%)** |
| --- | --- | --- | --- | --- | --- |
| *Capnopsis schilleri balcanica* | N/A | 0 | *Taeniopteryx nebulosa* | CROPL269-21 | 17.83 |
| *Zwicknia bifrons* | 0.71 | 0.92 | *Zwicknia rupprechti* | CROPL011-21 | 8.95 |
| *Zwicknia rupprechti* | 0.15 | 0.15 | *Zwicknia bifrons* | CROPL017-21 | 8.95 |
| *Siphonoperla taurica* | N/A | 0 | *Siphonoperla torrentium* | CROPL331-21 | 11.46 |
| *Siphonoperla torrentium* | 0.15 | 0.15 | *Siphonoperla taurica* | CROPL207-21 | 11.46 |
| *Xanthoperla apicalis* | N/A | 0 | *Siphonoperla torrentium* | CROPL331-21 | 17.04 |
| *Leuctra albida* | 1.55 | 2.64 | *Leuctra mortoni* | CROPL199-21 | 1.85 |
| *Leuctra bronislawi* | 0.15 | 0.15 | *Leuctra major* | CROPL082-21 | 13.09 |
| *Leuctra* cf. *inermis* | N/A | 0 | *Leuctra inermis* | CROPL201-21 | 3.59 |
| *Leuctra* cf. *prima* | 0.72 | 1.07 | *Leuctra prima* | CROPL023-21 | 9.28 |
| *Leuctra cingulata* | N/A | 0 | *Leuctra major* | CROPL082-21 | 5.9 |
| *Leuctra digitata* | N/A | 0 | *Leuctra* sp. | CROPL200-21 | 4.58 |
| *Leuctra fusca* | 1.01 | 3.94 | *Leuctra albida* | CROPL057-21 | 2.96 |
| *Leuctra hippopus* | 1.11 | 2.17 | *Leuctra* cf. *prima* | CROPL326-21 | 13.35 |
| *Leuctra hirsuta* | N/A | 0 | *Leuctra digitata* | CROPL136-21 | 15.36 |
| *Leuctra inermis* | N/A | 0 | *Leuctra* cf*. inermis* | CROPL130-21 | 3.59 |
| *Leuctra major* | 0 | 0 | *Leuctra cingulata* | CROPL135-21 | 5.9 |
| *Leuctra mortoni* | N/A | 0 | *Leuctra albida* | CROPL004-21 | 1.85 |
| *Leuctra nigra* | 0.16 | 0.46 | *Leuctra inermis* | CROPL201-21 | 13.01 |
| *Leuctra prima* | 1.13 | 1.38 | *Leuctra* cf*. prima* | CROPL326-21 | 9.28 |
| *Leuctra rauscheri* | 2.01 | 2.01 | *Leuctra* cf*. inermis* | CROPL130-21 | 5.9 |
| *Leuctra* sp. | 0.39 | 0.92 | *Leuctra mortoni* | CROPL199-21 | 3.92 |
| *Leuctra* sp*.* Z | N/A | 0 | *Leuctra prima* | CROPL003-21 | 14.16 |
| *Amphinemura standfussi* | N/A | 0 | *Protonemura praecox* | CROPL324-21 | 16.75 |
| *Amphinemura sulcicollis* | N/A | 0 | *Amphinemura triangularis* | CROPL153-21 | 12.16 |
| *Amphinemura triangularis* | 1.13 | 1.54 | *Amphinemura sulcicollis* | CROPL029-21 | 12.16 |
| *Nemoura avicularis* | 0.51 | 0.76 | *Nemoura sciurus* | CROPL067-21 | 11.14 |
| *Nemoura* cf. *rivorum* | 0 | 0 | *Nemoura flexuosa* | CROPL070-21 | 0 |

| **Species** | **Mean intra-Sp (%)** | **Max intra-Sp (%)** | **Nearest species** | **NN** | **Distance to NN (%)** |
| --- | --- | --- | --- | --- | --- |
| *Nemoura cinerea* | 1.42 | 2.17 | *Nemoura dubitans* | CROPL021-21 | 13.53 |
| *Nemoura dubitans* | 0.56 | 1.07 | *Nemoura sciurus* | CROPL091-21 | 9.94 |
| *Nemoura flexuosa* | 0.1 | 0.15 | *Nemoura* cf*. rivorum* | CROPL076-21 | 0 |
| *Nemoura marginata* | 2.94 | 4.58 | *Nemoura minima* | CROPL164-21 | 10.33 |
| *Nemoura minima* | N/A | 0 | *Nemoura marginata* | CROPL032-21 | 10.33 |
| *Nemoura mortoni* | N/A | 0 | *Nemoura minima* | CROPL164-21 | 10.51 |
| *Nemoura sciurus* | 0.33 | 0.61 | *Nemoura dubitans* | CROPL155-21 | 9.94 |
| *Nemoura uncinata* | N/A | 0 | *Nemoura minima* | CROPL164-21 | 13.27 |
| *Nemurella picteti* | 0.67 | 1.23 | *Nemoura flexuosa* | CROPL190-21 | 16.85 |
| *Protonemura auberti* | 0.98 | 2.49 | *Protonemura hrabei* | CROPL345-21 | 12.71 |
| *Protonemura* cf. *autumnalis* | N/A | 0 | *Protonemura hrabei* | CROPL069-21 | 8.73 |
| *Protonemura hrabei* | 0.41 | 0.46 | *Protonemura nitida* | CROPL161-21 | 7.21 |
| *Protonemura intricata* | 0.87 | 1.23 | *Protonemura hrabei* | CROPL069-21 | 14.69 |
| *Protonemura nitida* | 0.08 | 0.15 | *Protonemura hrabei* | CROPL069-21 | 7.21 |
| *Protonemura praecox* | 1.69 | 2.97 | *Protonemura hrabei* | CROPL345-21 | 13.23 |
| *Agnetina elegantula* | N/A | 0 | *Marthamea vitripennis* | CROPL355-21 | 16.91 |
| *Dinocras megacephala* | 0.81 | 1.7 | *Marthamea vitripennis* | CROPL355-21 | 21.29 |
| *Marthamea vitripennis* | N/A | 0 | *Agnetina elegantula* | CROPL268-21 | 16.91 |
| *Perla burmeisteriana* | N/A | 0 | *Perla illiesi* | CROPL313-21 | 0.15 |
| *Perla carantana* | 0.46 | 0.46 | *Perla pallida* | CROPL204-21 | 7.96 |
| *Perla illiesi* | 0.46 | 0.46 | *Perla burmeisteriana* | CROPL247-21 | 0.15 |
| *Perla marginata* | 0.57 | 1.08 | *Perla* sp. | CROPL245-21 | 0 |
| *Perla pallida* | 2.64 | 5.25 | *Perla marginata* | CROPL340-21 | 0 |
| *Perla* sp. | 0.3 | 0.3 | *Perla marginata* | CROPL287-21 | 0 |
| *Besdolus imhoffi* | 0.49 | 0.92 | *Perlodes microcephalus* | CROPL145-21 | 18.75 |
| *Isoperla albanica* | 0.61 | 0.61 | *Isoperla bosnica* | CROPL068-21 | 6.6 |
| *Isoperla bosnica* | 0.24 | 0.46 | *Isoperla grammatica* | CROPL205-21 | 3.45 |
| *Isoperla* cf. *lugens* | 0.76 | 2.16 | *Isoperla rivulorum* | CROPL314-21 | 6.73 |

| **Species** | **Mean intra-Sp (%)** | **Max intra-Sp (%)** | **Nearest species** | **NN** | **Distance to NN (%)** |
| --- | --- | --- | --- | --- | --- |
| *Isoperla goertzi* | 0 | 0 | *Isoperla bosnica* | CROPL068-21 | 7.83 |
| *Isoperla grammatica* | 1.29 | 3.44 | *Isoperla bosnica* | CROPL068-21 | 3.45 |
| *Isoperla illyrica* | 3.84 | 6.4 | *Isoperla tripartita* | CROPL122-21 | 0.15 |
| *Isoperla inermis* | 2.28 | 4.4 | *Isoperla popijaci* | CROPL232-21 | 11.96 |
| *Isoperla rivulorum* | 1.02 | 1.54 | *Isoperla* cf*. lugens* | CROPL354-21 | 6.73 |
| *Isoperla popijaci* | 0.04 | 0.15 | *Isoperla rivulorum* | CROPL306-21 | 6.74 |
| *Isoperla tripartita* | 2.04 | 6.57 | *Isoperla illyrica* | CROPL329-21 | 0.15 |
| *Perlodes dispar* | 0.46 | 0.46 | *Perlodes microcephalus* | CROPL145-21 | 8.46 |
| *Perlodes intricatus* | 0.46 | 0.46 | *Perlodes microcephalus* | CROPL145-21 | 10.93 |
| *Perlodes microcephalus* | 2.42 | 4.1 | *Perlodes dispar* | CROPL187-21 | 8.46 |
| *Brachyptera monilicornis* | 0.5 | 0.77 | *Brachyptera seticornis* | CROPL049-21 | 14.03 |
| *Brachyptera risi* | 0.3 | 0.76 | *Brachyptera monilicornis* | CROPL284-21 | 15.53 |
| *Brachyptera seticornis* | 1.43 | 2.17 | *Brachyptera monilicornis* | CROPL284-21 | 14.03 |
| *Brachyptera tristis* | 0.75 | 1.38 | *Brachyptera monilicornis* | CROPL065-21 | 17.65 |
| *Rhabdiopteryx acuminata* | 0.64 | 0.92 | *Zwicknia bifrons* | CROPL283-21 | 17.76 |
| *Taeniopteryx hubaulti* | N/A | 0 | *T. schoenemundi* | CROPL184-21 | 7.23 |
| *Taeniopteryx nebulosa* | 0.57 | 0.92 | *T. schoenemundi* | CROPL184-21 | 8.75 |
| *Taeniopteryx schoenemundi* | 1.13 | 1.54 | *Taeniopteryx hubaulti* | CROPL316-21 | 7.23 |
| *Taeniopteryx* n.sp. CRO-1 | 0.90 | 0.12 | *T. schoenemundi* | CROPL184-21 | 7.10 |
